# Bacterioplankton dynamics during winter freezing in a meltwater pond near Bratina Island, Antarctica

**DOI:** 10.3389/fmicb.2025.1707790

**Published:** 2026-01-14

**Authors:** Stephen E. Noell, Stephen D. J. Archer, Ian Hawes, S. Craig Cary, Ian R. McDonald

**Affiliations:** 1 Te Aka Mātuatua—School of Science, Te Whare Wānanga o Waikato—University of Waikato, Hamilton, New Zealand; 2 International Centre for Terrestrial Antarctic Research, University of Waikato, Hamilton, New Zealand

**Keywords:** Antarctica, archaea, meltwater ponds, microbial diversity, seasonal changes

## Abstract

The Bratina Island meltwater ponds, on the Ross Ice Shelf in Antarctica, undergo an annual freeze thaw cycle that results in progressive, extreme changes to the physical and chemical environments of the ponds. Here, we present the first investigation of the microbial community changes during this period using 16S rRNA gene sequence data from across the water column of Legin Pond, a stratified meltwater pond, from four time points that span the autumnal freeze period (January to April 2008). We found that the microbial community changed with the onset of winter, although water column depth and conductivity were also important factors influencing the community composition. We discovered a dominant presence of ASVs from the poorly characterized archaeal phylum “*Nanoarchaeota*” (now *Nanobdellota*), with abundance increasing with the onset of winter up to 95% of the total community at the final time point. Conversely, we observed a decrease over time in presumed aerobic, chemoorganotrophic groups from the phyla *Bacteroidota*, *Actinomycetota*, and *Pseudomonadota* (especially the *Alphaproteobacteria* class *Paracoccaceae*). Combined with previous complimentary physicochemical observations, our results paint a picture of a pond shifting from a mixed-layer community (part low-salinity enigmatic *Archaea*, part saltwater aerobic chemoorganotrophs) to a predominantly highly saline *Archaea* community that may have relied on heterotrophy to survive as the last of the pond water froze with the onset of winter.

## Introduction

Antarctic meltwater ponds are small, stable bodies of water that are formed from snow/ice melt that accumulates in depressions in the terrain and may be ephemeral or present for years. On annual cycles, some ponds thaw to the bottom in the summer only to freeze solid again during the winter (Schmidt et al., 1991; Hawes et al., 1999). The sea ice adjacent to Bratina Island, which is located at the northern tip of Brown Peninsula in the Ross Ice Shelf (Cowan and Tow, 2004), has a cluster of over 40 mapped and geochemically distinct ponds that have been the topic of research for several decades (De Mora et al., 1994). During autumn (between late January and April in Antarctica), light levels start reducing from the constant sunlight experienced during the summer, average air temperatures drop below zero and downwards ice growth in ponds proceeds, with accompanying salt exclusion raising salinity, until the pond is completely frozen (Hawes et al., 2011b). These ponds represent fascinating places to study how microbial life responds to massive, seasonal changes in the environment, as the dominant source of microbial biomass in the ice-free regions of Antarctica (Bottos et al., 2014).

Antarctic meltwater ponds support diverse microbial populations, since liquid water is not a limiting factor during the summer period as it is for Antarctic soils (Hawes et al., 2014). The bottoms of these ponds are often dominated by dense, perennial cyanobacterial mats, within which diverse protozoa and microinvertebrates are found. In the water column, there can also be a significant planktonic community made up of prokaryotes, photosynthetic flagellates, and heterotrophic protozoa (James et al., 1995; Sjöling and Cowan, 2003). Benthic microbial communities in these ponds tend to contain diverse *Bacteroidetes* and *Proteobacteria*, with evidence for potential aeolian dispersal between ponds (Archer et al., 2014, 2015, 2019). During the winter, less is known about bacterial activity and the microbial communities present, although most bacteria are believed to go into dormant states when the ice is frozen to protect themselves (Chattopadhyay, 2000; Foreman et al., 2011). Past studies have shown that the photosynthetic mats remain physiologically active as long as there is still liquid water, so deeper ponds shield microbial mats from the elements for longer and so remain active for longer (Hawes et al., 1999).

Most studies of meltwater ponds are limited to the Antarctic summer, as conditions later in the year are too extreme for safe sampling and movement throughout the continent. However, as part of the 2007–2008 International Polar Year, samples were collected from several meltwater ponds around Bratina Island for an extended period that encompassed the beginning of the annual freeze and the passage from 24 h of daily irradiance to near darkness (late January to early April). This comprehensive project covered changes to the physical environment (ice cover, irradiance, conductivity), chemistry (pH, chemical species, ion extrusion), and biological activity (grazing, autotrophy, heterotrophy, etc.) (Hawes et al., 2011a; Hawes et al., 2011b; Safi et al., 2012; Webster-Brown et al., 2012).

Past results published from this “extended season” project showed that, as pond water froze, the salts in the pond water became excluded from the ice and were concentrated in the liquid water (Hawes et al., 2011a). This resulted in a very concentrated brine, with many minerals (calcite, Fe, Mo, Cu, and Zn) precipitating out of the water column during the freezing process (Webster-Brown et al., 2012). Interestingly, as the amount of liquid water shrank during this transition, photosynthesis continued unabated in the remaining unfrozen benthic cyanobacterial mats, resulting in supersaturating dissolved oxygen concentrations at some depths and high pH (>8.5) due to the blockage of gas exchange with the atmosphere by ice cover (Hawes et al., 2011b). When photosynthesis began to slow down as the transition to winter darkness proceeded, the pH in the water slowly dropped again and inorganic carbon accumulated (Hawes et al., 2011b). Eventually, the remaining liquid layer is thought to become a super-concentrated, anoxic brine as this oxygen is depleted (Foreman et al., 2011; Hawes et al., 2011b), with liquid brine temperatures of close to −20 °C recorded at the end of winter (Wait et al., 2006). In terms of biological activity, during the freezing transition, microbial abundance decreased markedly, driven in part by grazing pressure (Safi et al., 2012). Autotrophs tended to decrease in abundance more than heterotrophs, leaving a primarily heterotrophic-driven ecosystem in the remaining liquid layer at the end of autumn (Safi et al., 2012).

One of the key components that was lacking from these past studies published as part of this “extended season” project was taxonomic identification of the microorganisms present in these meltwater ponds and how individual groups respond to the onset of winter and associated chemical changes. This study aims to fill that gap using 16S rRNA gene sequencing data from one of the stratified ponds studied, Legin Pond. Samples from four time points over this autumn transition were sequenced, across multiple depths. Our hypotheses were two-fold: (1) that the changes in the chemical and physical nature of the pond over the onset of winter would result in a shift in the microbial community present, with depth also playing a role in influencing microbial community composition given the stratified nature of Legin Pond; (2) that the decreased sunlight with the onset of winter would result in a shift from photoautotrophy to heterotrophy over time.

## Materials and methods

### Extended season sampling

**Figure 1:**
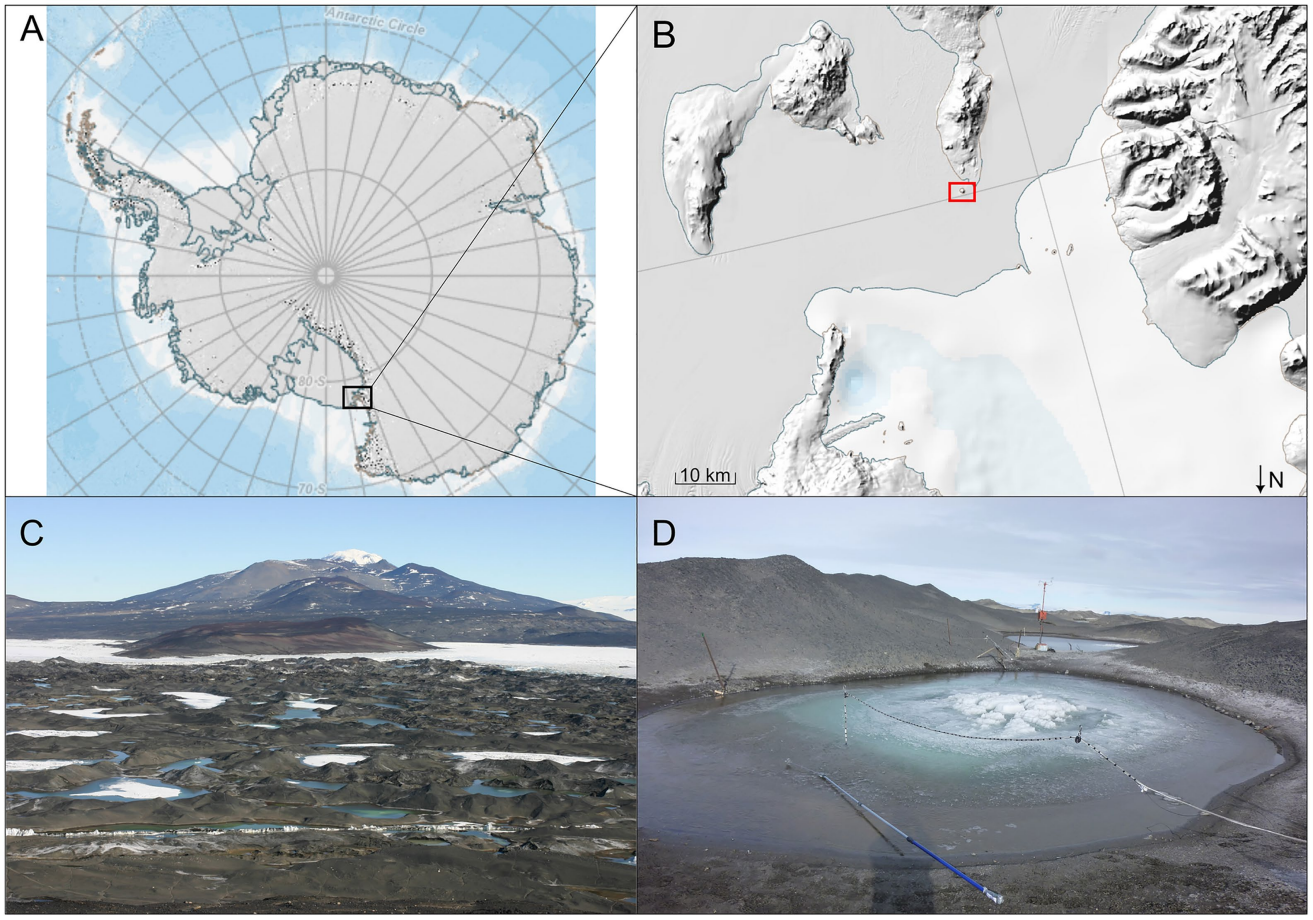
Map and images of Bratina Island and setting. **(A)** Map of Antarctica, with region of interest in the Ross Ice Shelf/Ross Sea region in the black box. **(B)** Zoomed in region from **A**, showing the edge of Ross Island, the Ross Ice Shelf, and the Ross Sea, with Bratina Island highlighted in the box. **(C)** Image taken from Bratina Island, showing the surrounding meltwater ponds to the south, with Brown Island in the background. **(D)** Image showing Legin Pond and sampling apparatus. Maps in **A,B** are from the Antarctic Digital Database Map Viewer https://www.add.scar.org/, Open Source. Images in **C,D** are courtesy of Ian McDonald.

Details of the study site, sampling methods and physical characteristics have been described previously in Hawes et al. (2011a). In brief, near the northern tip of Brown Island peninsula, Antarctica (Latitude 78°01′S, Longitude 165°32′E) lie three huts established as a base from which the adjacent ponds can be studied (see Figure 1). Samples for this study were collected from a small pond nearby (unofficial name of Legin Pond) on the Ross Ice Shelf from early January to early April 2008. Dissolved oxygen, temperature and conductivity measurements were collected using probes attached to a graduated pole at various depths within the pond throughout the study period. Data were recorded against depth during downwards profiling but, because of changes in overall pond depth due to freezing, were subsequently converted to distance from the pond bottom as in Hawes et al. (2011a).

At four time points (13 February, 22 February, 11 March and 3 April) samples of Legin water at different depths were filtered through syringe-mounted 35 mm 0.22-μm filters (Whatman International Ltd., Kent, United Kingdom) until the filter clogged (between 3 and 200 mL, due to the ease of filtration and as a rough proxy for similar biomass levels) to concentrate biomass. Filters were preserved using CTAB buffer for analysis at the University of Waikato. Geochemical and physical data used in this paper comprise a mix of previously published data (Hawes et al., 2011a) and unpublished data supplied by Hawes and Safi. This information was combined with the results of DNA sequencing experiments at the University of Waikato.

### DNA extraction of samples

DNA was extracted from filtered water samples using a modified CTAB extraction protocol (Dempster et al., 1999). Frozen, sealed 0.22 μm sample filters were first thawed on ice and then connected to a syringe containing 1 mL of fresh CTAB. The exposed filter nipple was parafilmed and the entire assembly, including the whole filter, was incubated in a Ratek Orbital mixer at 150 rpm and 65 °C for 30 min. The filter assembly was allowed to cool then 0.5 mL of the CTAB in the syringe was pushed through the filter assembly to evacuate the lysate in an Eppendorf tube. An equal volume of chloroform/isoamyl alcohol (24.1) was then added to the lysate and mixed on the orbital mixer at 150 rpm and 65 °C for a further 30 min. The Eppendorf tube was then centrifuged for 15 min at 12,500 rpm, and 0.2 to 0.5 mL of the aqueous phase (as determined by incremental 100 μL transfers from the Eppendorf autopipette, depending on the size of the interphase and aqueous phase) was transferred to a new tube. To initiate nucleic acid precipitation 1 volume of isopropanol and 0.5 volume of 10 M NaCl was added and then incubated at −80 °C for at least one hour. The tube was then centrifuged at 15,500 rpm for 30 min, the supernatant discarded, the DNA pellet washed with 0.5 mL of 70% EtOH and then centrifuged at 15,500 rpm for 5 s. The pellet was dried and resuspended in 10–50 μL of sterile milliQ H_2_O depending on the visibility and size of the pellet to optimise low biomass samples. The extracted DNA was initially quantified using a Nanodrop ND-1000 at 260 nm (NanoDrop Technologies, Montchanin, DE) and then frozen at −80 °C until further analysis.

### Illumina sequencing

Prior to PCR amplification, DNA in previously frozen DNA samples was quantified using a fluorometer (Denovix DS-11FX+). The 16S rRNA gene (V4–V5 region) was amplified using PCR with the 515YF and 926R primers (Caporaso et al., 2018; Ul-Hasan et al., 2019), adapted for Illumina sequencing using fusion primers with a unique tag on the forward primer (Comeau et al., 2017). As described in the Comeau paper, the use of fusion primers allows for a single PCR run to be conducted instead of the usual two-step PCR method for Illumina sequencing (primer-specific PCR followed by indexing PCR with Illumina primers), reducing chances for error, production of chimeras, and costs.

The sequences used for these primers are as follows. For the 515YF fusion primers, from 5′ to 3′: left arm P5 adapter: AATGATACGGCGACCACCGAGATCTACAC, unique Nextera XT v2 i5 barcode (8 bases), right arm P5 adapter: TCGTCGGCAGCGTCAGATGTGTATAAGAGACAG, 515YF forward primer (Parada): GTGYCAGCMGCCGCGGTAA. For the 926R fusion primers, from 5′ to 3′: left arm P7 adapter: CAAGCAGAAGACGGCATACGAGAT, Nextera XT v2 i7unique barcode (8 bases), right arm P7 adapter: GTCTCGTGGGCTCGGAGATGTGTATAAGAGACAG, 926R reverse primer (Quince): CCGYCAATTYMTTTRAGTTT. The 20 μL reaction mixture included 0.24 mM dNTPs, 1.2 × PCR buffer, 6 mM MgCl_2_, 0.016 mg/mL BSA, 0.2 mM of each primer, 0.024 U Taq polymerase (Thermo Fisher Scientific, Massachusetts, United States), and 9 ng of genomic DNA.

The PCR reaction conditions were: initial denaturation (94 °C), 3 min; 30 cycles of 94 °C for 45 s, 50 °C for 1 min, and 72 °C for 1.5 min; final extension was 72 °C for 10 min. All PCR reactions were run on an Applied Biosystems ProFlex PCR System (Thermo Fisher Scientific). PCR reactions were run in triplicate to account for possible PCR bias. PCR was also conducted on negative (extraction, process, and reagent) and positive (extracted DNA from New Zealand soils) controls interspersed among the samples. Quality and absence of amplification in negative controls were checked using electrophoresis gel and successful amplicons were quantified via fluorometer (Denovix DS-11FX+).

Following pooling of triplicate PCR products from each sample, 25 μL of each sample was treated with Invitrogen SequalPrep Normalization (Thermo Fisher Scientific) to purify, normalize the PCR product concentration (to 0.4 ng/μL), and remove DNA fragments smaller than 100 bp (Comeau et al., 2017). Samples were eluted in 50 μL volume after PCR product purification and concentration checked via fluorometer (Denovix DS-11FX+). The Illumina amplicon library was constructed using 2 μL from each purified PCR product and quantified via fluorometer (Denovix DS-11FX+). 16S rRNA gene sequencing was conducted on all samples on a MiSeq v3 Illumina sequencer at Massey Genomic Services (Palmerston North, NZ), with quality checked via Agilent Bioanalyzer with the High Sensitivity Assay prior to sequencing.

### DNA sequencing data processing

DNA sequences were demultiplexed and primers and tags removed by the sequencing provider using imbedded i5/i7 tags provided in the NEXTERA v2 format. Residual primers were trimmed using cutadapt (Martin, 2011). Subsequent data analysis was conducted using R v. 4.4.2. Illumina sequence data raw reads were processed using the DADA2 pipeline with default parameters, v. 1.30.0 (Callahan et al., 2016). Chimeras were removed using the function “removeBimeraDenovo,” method “consensus.” The ASVs were assigned taxonomy using the SILVA database No. 99 v138.2 (McLaren, 2024). Taxonomy changes rapidly in the field of microbiology, resulting in some of the taxonomic names used in this version of the SILVA database being outdated. However, we have chosen to leave the SILVA taxonomy in place in the data and figures but have noted updated taxonomy for relevant taxa in the text. Sequences unassigned at the Domain level, assigned to Eukaryota, mitochondria, and chloroplast were removed using the following commands:     “physeq <- subset_taxa (physeq, !is.na(Kingdom) | (Kingdom !=“Eukaryota”))
     physeq <- subset_taxa (physeq, is.na(Order) | Order !=“Chloroplast”)
     physeq <- subset_taxa (physeq, is.na(Family) | Family != “Mitochondria”)”


ASVs with less than 10 reads in all samples were removed (low abundance ASVs, 90). The R-packages Decipher (Wright, 2016) and phangorn (Schliep, 2011) were used to generate an unrooted phylogenetic tree by the neighbor-joining method. Following ASV generation, additional chimeras were removed using:“vsearch --uchime_ref es_illumina_trimmed_ASVs.fasta --db SILVA_138.2_SSURef_NR99_tax_silva.fasta --uchimeout uchime_out.txt”


The resulting set of ASVs was used for all subsequent analyses, except Supplementary Figure S1 (showing chloroplast abundance distribution). The taxonomy of these ASVs were manually inspected for the presence of common reagent contaminants (Laurence et al., 2014; Salter et al., 2014; de Goffau et al., 2018), but none were found.

### ASV data analysis

All plots were visualized using ggplot2 v. 3.5.1 (Wickham, 2016) with beautification of figures in Inkscape.^1^

To assess alpha diversity of samples, raw ASV read counts were first rarefied to an even sequencing depth (sample with the lowest read count was used as the read count for rarefaction, 7,044 reads) using the phyloseq v. 1.46.0 (McMurdie and Holmes, 2013) function “rarefy_even_depth.” Alpha diversity was assessed within vegan v. 2.6-8 (Oksanen et al., 2020) via the Shannon index. Rarefied sequence counts were only used for alpha diversity analysis. Unconstrained beta diversity of the microbial community structures was investigated by transforming raw sequence abundances to relative abundance using the phyloseq function “transform_sample_counts,” then calculating a Unifrac distance matrix using phyloseq. This Unifrac distance matrix was used to perform PERMANOVA and PERMDISP tests on the microbial community composition using different independent variables with the “adonis2” (for PERMANOVA) or “betadisper” followed by “permutest” commands within the vegan package.

To assess correlations between microbial community composition (using ASV counts transformed via relative abundance), we conducted several tests. First, environmental factors correlating with microbial community composition were identified using mantel tests within microeco v. 1.10.0 (Liu C. et al., 2021), with Bray–Curtis used as the method for calculating community dissimilarity matrix and Pearson used to calculate correlations between matrices, with Benjamini–Hochberg corrected *p*-values. The environmental factors that correlated significantly (*p* < 0.05, Benjamini–Hochberg correction) with microbial community structure were plotted using a distance-based redundancy analysis (dbRDA) within microeco (“plot_ordination”), using relative abundance normalized ASV counts. To identify correlations between specific taxa and environmental factors, we used a redundancy analysis (RDA) (“cal_ordination, method = ‘RDA’”) at the phylum or genus level within microeco, plotted using “plot_ordination,” using relative abundance transformed ASV counts. Correlations between taxa (relative abundances) and environmental factors were calculated within microeco using a spearman’s correlation within the “cal_cor” function with a Benjamini–Hochberg correction across all data (“p_adjust_type = ‘All’”). The resulting heatmap was plotted using “plot_cor” within microeco. Additional clustered heatmaps were generated using the pheatmap R package v. 1.0.12 (Kolde, 2019).

## Results

### Physicochemical analysis

Detailed information on changes in the physical, chemical and biological variables that occurred in Legin pond during this time period can be found in the “Summer-winter transitions in Antarctic ponds” paper series (Hawes et al., 2011a; Hawes et al., 2011b; Safi et al., 2012; Webster-Brown et al., 2012). In this paper, we have classified the four time points that were taken based on the ice thickness of Legin Pond at the time of sampling, to give an indication of the season in which the sample was taken (Supplementary Table S1).

**Figure 2:**
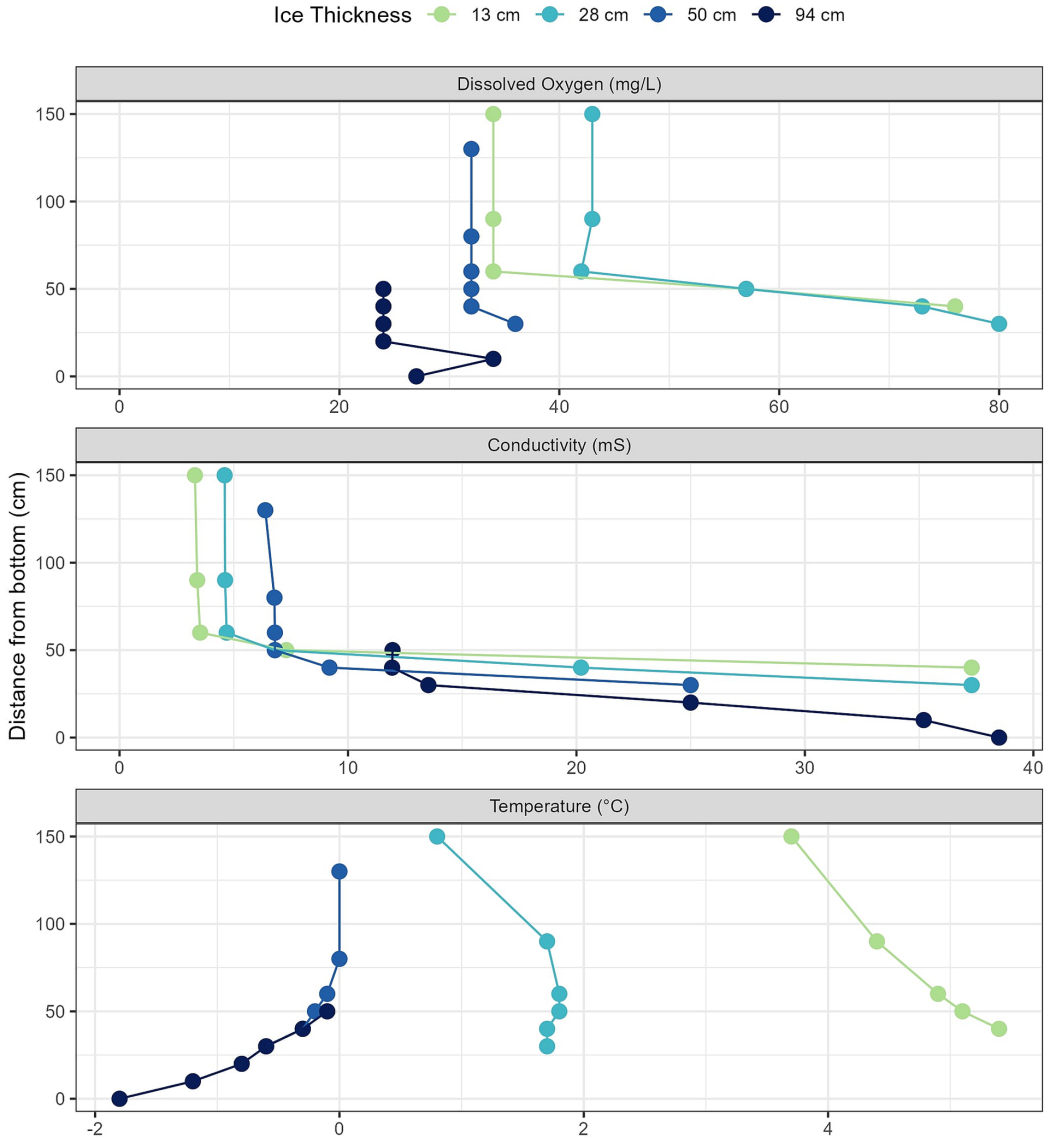
Depth profile from Legin Pond across four time points between February and April 2008 where the ice was of increasing thickness on top of the pond. Data was re-plotted from Hawes et al. (2011a).

In brief, we observed that the pond water columns were density stratified from the outset of sample collection. All profiles showing an upper mixed layer (mixolimnion) with increasing conductivity over time, and a lower, more saline water layer (chemocline) at and below 50 cm above sediment level (Figure 2, middle panel). All chemocline samples had conductivity >20 mS. Water temperatures followed predictable patterns across time points, with decreasing temperature later in the season (Figure 2, lower panel). At the last two time points, samples from all depths were at or below 0 °C with basal temperature approaching −2 °C by the end of the observation period. As the ice thickened, the dissolved oxygen (DO) concentrations increased in the more saline basal layer (Figure 2, top panel). DO reached super-saturated concentrations at almost all samples of the depth profiles in the first two time points, with DO exceeding 50 mg/L, max 80 mg/L (Supplementary Table S1). The latter two time points, however, had maximal concentrations of 37 mg/L (Supplementary Table S1), although these concentrations were still well above atmospheric oxygen concentrations.

### High throughput sequencing

To examine the changes in microbial communities in Legin Pond during the transition from summer to autumn, we conducted Illumina sequencing of 23 water samples. This generated a dataset of 941,914 reads after ASV calling and chimera removal (read tracking through the pipeline is available in Supplementary Table S2), which consisted of 296 ASVs after removal of low abundance ASVs and ASVs assigned to Eukaryotes, mitochondria, and chloroplasts. We chose to remove these mitochondrial and chloroplast ASVs as taxonomy of eukaryotic organisms can be challenging to infer from chloroplast or mitochondrial 16S sequences. However, prior to removing these mitochondria and chloroplast ASVs but after removing low abundance ASVs, we did observe that chloroplast (44 ASVs) as well as one *Cyanobacteriota* ASV decreased in abundance over time, with all photosynthesizers almost entirely absent at the last time point (Supplementary Figure S1). These chloroplast ASVs made up 17% of the total reads in the dataset, with a maximum relative abundance of 53% in the first time point, 60 cm from bottom of the pond sample (Supplementary Figure S1).

In our trimmed data set (low abundance, eukaryotic, mitochondria, and chloroplasts removed), we found that the 5 most abundant ASVs accounted for 43% of all reads; all these ASVs came from the “*Nanoarchaeota*” phylum [now *Nanobdellota*, (Göker and Oren, 2023)], class “*Nanoarchaeia*” (Supplementary Table S3). All except two of these were classified to the order *Candidatus* Woesearchaeales; the others were classified to the order *Candidatus* Pacearchaeales, with no family assigned to any. In total, we found 120 ASVs from the “*Nanoarchaeia*” class (41% of all ASVs).

We observed that all sampling time points had similar median read counts per sample, although there was a wide range in diversity in the number of reads per sample (Supplementary Figures S2A,B). We also observed that all samples reached sequencing saturation (Supplementary Figure S2C).

### Alpha and beta diversity and community composition

**Figure 3:**
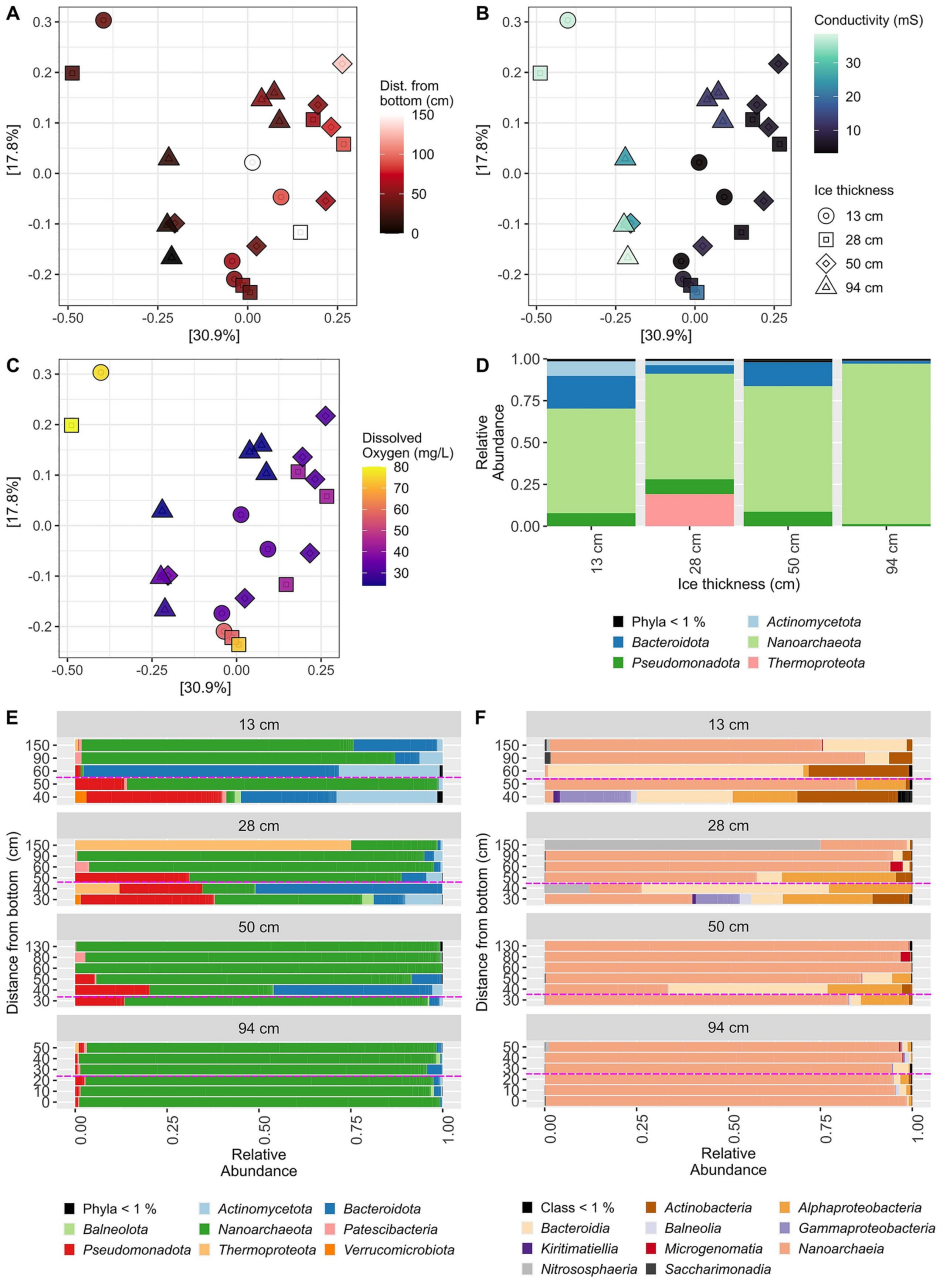
Biodiversity analysis of prokaryotic (16S rRNA gene) community across all four time points and depths in Legin Pond. **(A–C)** Beta diversity measured with a principal coordinates analysis (PCA) using UniFrac distances, with relative abundance normalized read counts for ASVs. The percentages next to the axes names indicate the amount of variation in the data that that axis explains. Samples are colored by **(A)** distance from the bottom of the pond, **(B)** conductivity, **(C)** or dissolved oxygen. **(D–F)** The relative abundance of prokaryotic groups, either phylum **(D,E)** or class **(F)**, at each time point, either **(D)** with all depths aggregated, or **(E,F)** with each depth separate. In **E,F**, the dashed line marks the boundary between mixolimnion (<20 mS conductivity, on the top) and chemocline (>20 mS conductivity, on the bottom).

After processing our DNA sequence data, we first asked whether there was any clear grouping of samples based on sampling time point and/or depth in the beta diversity analysis (Figure 3A). Notably, we did not see any clustering of samples by sampling time point. Instead, we found that the deepest samples primarily clustered on the left side of the graph, while the other depths were mixed together on the right side. This clustering was most apparent when conductivity was considered instead, with the most saline samples (also called the chemocline in Safi et al. 2012) also being the deepest samples (Figure 3B). These clustered away from the lower conductivity, or mixolimnion, samples, on the right side. There were two samples that separated from all others in this analysis: Legin_13.02-140_02 and Legin-150.22.02_58. These samples were both the bottom-most samples from the first two time points and were the only samples that had both high conductivity (37 mS) and super-saturated oxygen concentrations (>76 mg/L) (Figure 3C and Supplementary Table S1). It is feasible that these samples may have unintentionally included some of the pond sediment or even microbial mats known to underlie the water column, but it is difficult to know for certain.

We also found that grouping samples by “Ice thickness” (i.e., sampling date) did not provide a significantly different centroid between groups (PERMANOVA test, *p* = 0.192), while depth (i.e., distance from the bottom of the pond) did (PERMANOVA test, *p* = 0.009, *R*^2^ = 0.117), although the strength of correlation was weak. The strength of correlation increased when we divided the samples into “chemocline” or “mixolimnion” samples instead (PERMANOVA test, *p* = 0.001, *R*^2^ = 0.207). We also found non-significant results from a permutation test for homogeneity of multivariate dispersions with depth, indicating the significant PERMANOVA results for depth are not the result of differences in data dispersion between depths (*p* = 0.077). Thus, it seems that the stratified nature of Legin Pond is partially confounding any influences of sampling time on the microbial community. We also did not find significant differences between sampling time points in terms of alpha diversity using the Shannon index (Supplementary Figure S3).

Although we did not see clear clustering of samples by time point in the beta diversity analysis, we did observe some notable differences in phylum-level abundance between time points when all depths were aggregated (Figure 3B). Of note was the increase in “*Nanoarchaeota*” abundance, with ASVs from this phylum increasing from 49% in the first time point up to 96% relative abundance at the final time point. On the other hand, *Actinomycetota* (mainly class “*Actinobacteria*”, also known as *Actinomycetes*) and *Pseudomonadota* (mainly classes *Alphaproteobacteria* and *Gammaproteobacteria*; previously known as phylum *Proteobacteria*) both decreased in abundance from the first two time points to less than 2% abundance in the final time point. When broken down by depth, we observed some differentiation between mixolimnion and chemocline samples (Figure 3C). We observed that *Pseudomonadota* and *Bacteroidota* were primarily found at lower depths, while *Thermoproteota* were mainly found near the surface.

We looked more closely at the abundance profiles of “*Nanoarchaeota*” ASVs, given their dominance in this pond, and found that abundance profiles distinguished two groups of “*Nanoarchaeota*” ASVs (Supplementary Figure S4). One group was only found in samples with conductivity >20 mS (with the exception of two samples from the first two time points that were taken from the chemocline), while the other group was almost exclusively found in samples <20 mS. These two groups of ASVs may thus represent groups of “*Nanoarchaeota*” adapted to different levels of salinity; however, DO, temperature, or other unmeasured physicochemical parameters may also be playing a role in the abundances of these different groups.

### Correlations between biology and physicochemistry

**Figure 4:**
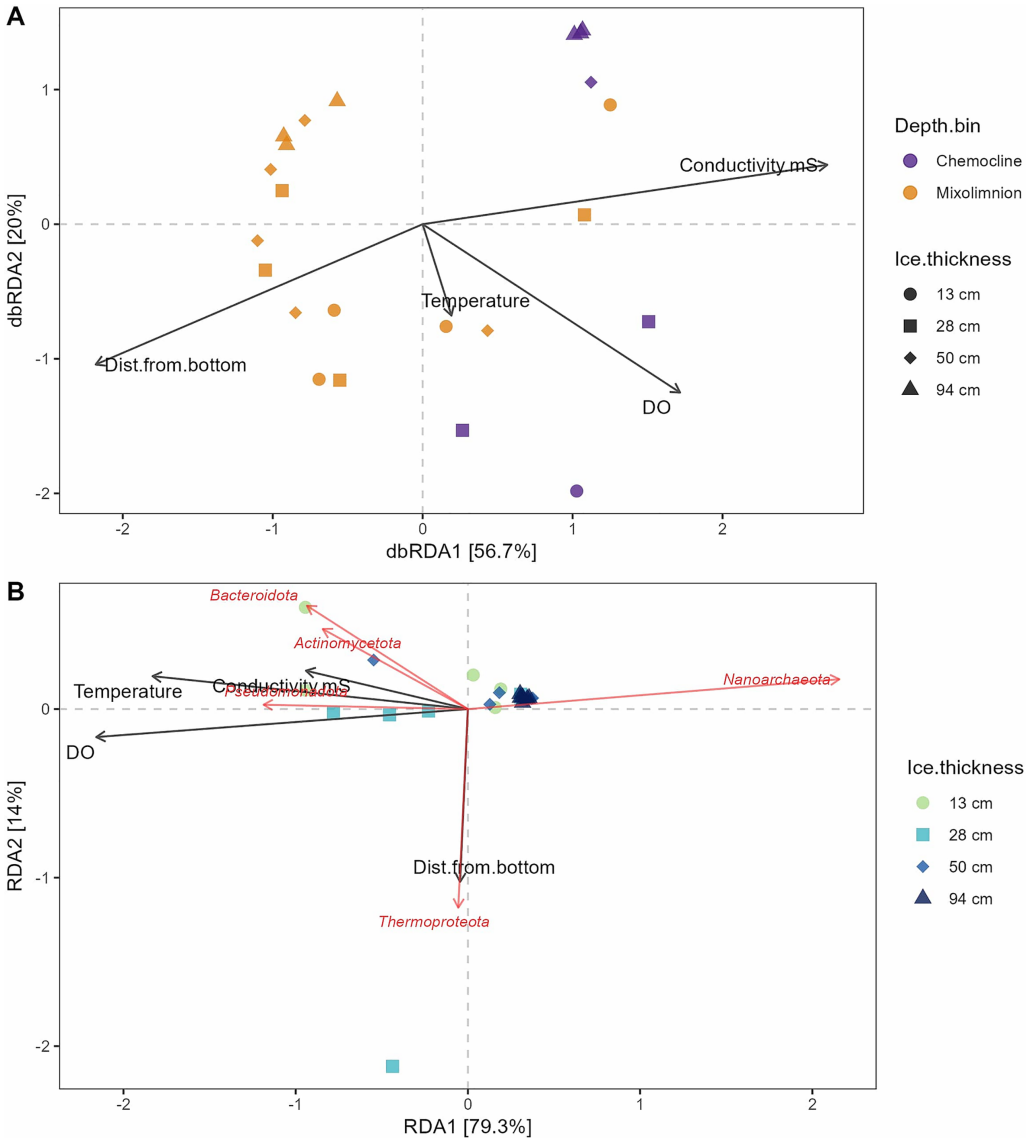
**(A)** Distance-based redundancy analysis of environmental factors that correlated with the prokaryotic communities (using relative abundance normalized read counts for ASVs) across sampling time points and depths. Environmental factors shown had a significant (*p* < 0.05) correlation with the communities based on a Mantel test after adjusting for multiple comparisons using a Benjamini–Hochberg adjustment (see Table 1). **(B)** Redundancy analysis of prokaryotic communities (using relative abundance transformed read counts for ASVs) with physicochemical factors and the five most abundant taxa at the phylum level incorporated as explanatory variables. DO, dissolved oxygen, mg/L; Dist.from.bottom, Distance from bottom of pond, cm.

**Figure 5:**
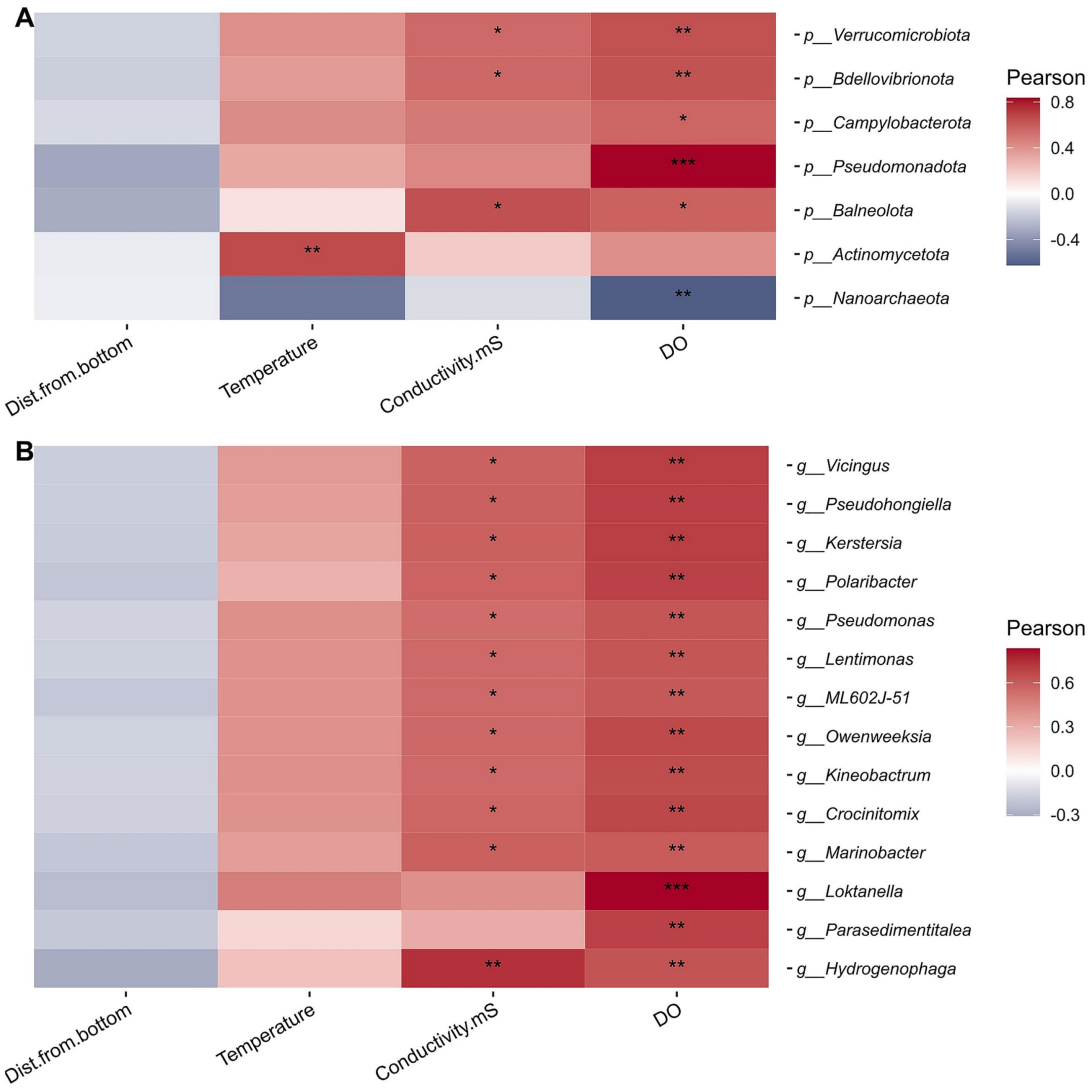
Spearman rank correlation heatmap of physicochemical parameters and prokaryotic **(A)** phyla or **(B)** genera (using relative abundance normalized read counts). DO, dissolved oxygen, mg/L; Dist.from.bottom, distance from bottom of pond, cm. The significance stars correspond to the following adjusted *p*-values (Benjamini–Hochberg correction): ^*^p = 0.05, ^**^p = 0.01, and ^***^p = 0.001.

We next asked whether there were any correlations between the physicochemistry of the samples and the microbial communities present (Figures 4, 5). We found that all four physicochemical parameters measured (dissolved oxygen, conductivity, depth, and temperature) were significantly correlated with microbial community structure (Table 1; Mantel test, adjusted *p*-value < 0.05, Benjamini–Hochberg correction). Conductivity had the largest correlation coefficient (0.320) of the four factors tested. These four factors alone were able to account for 77% of the variation in the microbial community structure (Figure 4A), although it is likely that other, unmeasured variables also have strong correlations.

**Table 1:** Results of Mantel tests to identify correlations between environmental parameters and microbial community structure (at the ASV level), using a Spearman correlation method and Benjamini–Hochberg adjustment for multiple tests, with ASV read counts normalized via relative abundance.

Variables	Correlation coefficient	Adjusted *p*-value
Dissolved oxygen (%)	0.226	0.011
Conductivity (mS)	0.320	0.008
Distance from bottom (cm)	0.200	0.018
Temperature (°C)	0.229	0.011

We found that samples in the top right corner, similar to Figures 3A–C, clustered together and had higher conductivity. Surface samples tended to cluster in the bottom left corner of the graph. Interestingly, in this analysis, the chemocline samples from the latter two time points separated out from the first two time points, correlated with higher DO and temperature. The two outlier samples that were noted from Figure 3C had strong correlations with the higher DO levels observed in those samples. However, we did also find that several environmental factors were significantly correlated with each other; temperature was significantly correlated with all three other factors, as well as conductivity and distance from pond bottom, as would be expected (Supplementary Table S4). Thus, autocorrelation might be a factor in these results.

Adding in microbial taxa at the phylum level increased the explanatory power of the RDA axes to 93% (Figure 4B). We found that the six samples with higher abundances of *Bacteroidota*, *Pseudomonadota,* and *Actinomycetota* tended to have larger dissolved oxygen concentrations, conductivity, and temperatures. We observed a tight cluster of samples on the right side of the graph that were distinguished by having large abundances of “*Nanoarchaeota*”, opposite to the warmer, saltier, higher DO samples. Interestingly, a single sample (Legin-150.22.02_58) ended up clustering away from all other samples in the RDA, distinguished by being a surface sample and having high abundances of *Thermoproteota*.

Correlations between taxa at the phylum level and physicochemical factors confirmed these results, with multiple phyla (including *Pseudomonadota*) having significant, positive correlations with DO and (to a lesser extent) conductivity, while “*Nanoarchaeota*” were significantly negatively correlated with dissolved oxygen (Figure 5A). *Actinomycetota* also had a significantly positive correlation with temperature. At the genus level, we observed a number of genera that had significant, positive correlations with DO and, often, conductivity (Figure 5B). All these genera were from the phyla *Bacteroidota* or *Pseudomonadota* (primarily, class *Gammaproteobacteria*). The strongest correlation of these was with the genus *Loktanella* from the class *Alphaproteobacteria*. Across all taxonomic levels, we did not observe any significant correlations with “distance from the bottom.”

## Discussion

The summer-to-winter transition in the meltwater ponds surrounding Bratina Island provide a fascinating opportunity to examine how rapidly microbial communities respond to drastic, seasonal shifts in light, nutrient availability, and chemistry. Our results provide a more rounded idea of the microbial community present in this pond, as opposed to previous reports of microbial diversity from meltwater ponds that come from a single time point, typically during the peak melt and biological activity in the austral summer. Here, we discuss our results in light of our two hypotheses.

### Hypothesis 1.1: influence of time on microbial community composition

As hypothesized, we did see changes in the microbial community between different time points; it is likely that the stratified nature of Legin Pond was in part responsible for the lack of significant PERMANOVA results. Most notably, we observed (Figure 3D) a loss of almost all microbial groups from the community other than ASVs from the “*Nanoarchaeota*” phylum (now *Nanobdellota*), which is part of the DPANN group. In particular, the order *Ca.* Woesearchaeales (also referenced as the *Woesearchaeota* phylum Adam et al., 2017; Parks et al., 2017), the most abundant order of this phylum in our data set, is known for conspicuous metabolic deficiencies. This indicates strong dependence on other microorganisms, potentially through syntrophic relationships (Wurch et al., 2016; Castelle et al., 2018; Liu et al., 2018; Beam et al., 2020; Sakai et al., 2022). Metagenomics of diverse members of this group have indicated that, in general, members of this group are anaerobic or facultative anaerobic heterotrophs, with strong associations with methanogens (Liu et al., 2018; Liu X. et al., 2021; Huang et al., 2021; Cloarec et al., 2024). Members of this order have been previously observed in aquatic, high-saline, occasionally anoxic environments globally (Liu et al., 2018; Liu X. et al., 2021; Pal et al., 2020; Qin et al., 2023; Cloarec et al., 2024). Legin Pond at the onset of winter nearly matches this description, with highly saline water and oxygen levels that likely eventually become anoxic based on results from other Bratina Island ponds (Hawes et al., 2011a; Hawes et al., 2011b; Webster-Brown et al., 2012). Additionally, sediment incubations from Bratina Island ponds reported that methanogenesis was an increasingly favoured electron pathway during winter conditions (Mountfort et al., 2003). Anoxic microzones in otherwise oxic water columns are common in Antarctic melt ponds and allow anaerobic processes to coexist with photosynthetic oxygen supersaturation (Priscu et al., 1998; Laybourn-Parry and Pearce, 2016).

Given that we only have access to 16S rRNA gene sequence data, it is impossible to know whether these “*Nanoarchaeota*” ASVs were alive at this final time point or, if alive, also metabolically active. One possible explanation for our results is that these cells were simply the last to lyse and have their DNA degraded. However, previous results (Safi et al., 2012) indicated that microbial productivity (as measured using the ^3^H-thymidine incorporation method) was high during the final sampling time point, even as microbial cell numbers became quite low. Thymidine incorporation only provides information about actively replicating microbes that have thymidine transporters; given that we do not have genomes or metagenomes for any of these “*Nanoarchaeota*” species, it is impossible to know whether they have thymidine transporters. However, they did comprise 96% of the microbial community present, so it is likely that the thymidine incorporation measured in this previous study was due to replication activity by “*Nanoarchaeota*” cells. However, if these “*Nanoarchaeota*” species are indeed syntrophic, it is unclear how they might be surviving at this last time point, when they dominate the planktonic community and larger eukaryotes are almost completely absent (Safi et al., 2012). It could be that they are thriving off the organic soup left behind by dead microbes, resulting in the previously observed stagnant DOC concentrations despite the reduction in pond volume (Safi et al., 2012). Further metagenomics, metatranscriptomics, and microscopy experiments are needed to validate this hypothesis.

Our results indicating large abundances of “*Nanoarchaeota*” are especially striking given that past studies of microbial diversity in Bratina Island meltwater ponds did not find many Archaea present: no Archaeal OTUs were among the 15 most abundant OTUs and *Euryarchaeota* (previous taxonomic classification for “*Nanoarchaeota*”) OTUs only made up a tiny percentage (<1%) of the community in a few ponds (Archer et al., 2014, 2015, 2016). This discrepancy is likely due to past studies using primers for a different region (V5-V6) of the 16S rRNA gene, advances in taxonomic classification software, time of sampling as previous results were during peak austral summer, and/or advancements in descriptions of this group of Archaea. Nonetheless, until now, it was unknown that the planktonic microbial communities present in Legin Pond, at least during these time points, were dominated by *Archaea*, not *Bacteria*.

Over time, we observed that ASVs from three bacterial phyla were almost entirely lost from the microbial community: *Bacteroidota*, *Pseudomonadota* (formerly *Proteobacteria*), and *Actinomycetota*. These bacterial groups have previously been found in large abundances in Bratina Island meltwater ponds during the austral summer (Archer et al., 2014, 2015, 2016). The most common genera from these phyla (*Algoriphagus* within *Bacteroidota*; *Yoonia*, *Loktanella*, *Pelagimonas*, and *Parasedimentitalea* from *Pseudomonadota*; and *Aquiluna* from *Actinomycetota*) have previously been isolated from marine and/or brackish lake systems and are known to be aerobic chemoorganotrophs (Lau et al., 2004; Kang et al., 2012; Park et al., 2014; Evtushenko, 2015; Nedashkovskaya and Vancanneyt, 2015; Pitt et al., 2021; Feng and Xing, 2023; Huang et al., 2024) (Figures 3C,D and Supplementary Table S3). Previously isolated *Aquiluna* strains are also capable of photoheterotrophy via actinorhodopsins (Kang et al., 2012; Pitt et al., 2021), a strategy that is unlikely to be successful in late autumn as sunlight becomes increasingly unavailable. Interestingly, members of the *Loktanella* genus previously have been isolated from microbial mats in Dry Valley lakes, relatively close to Bratina Island (Van Trappen et al., 2004).

It is not apparent what environmental factors are responsible for the loss of these microbial groups over time. The *Pseudomonadota* phylum, and multiple genera within this phylum, showed significant, positive correlations with DO and, in many cases, conductivity (Figure 5). However, DO levels remained aerobic even at the last time point, and maximum conductivity levels at the last time point were similar to those at other time points (Figure 2). It is possible that these microbial groups are tolerant of supersaturated oxygen levels and thus were lost as oxygen levels returned to simply saturated levels. It is also highly likely that other, unmeasured, environmental factors were responsible for the loss of *Pseudomonadota* and *Bacteroidota* ASVs. We did observe that their decline in abundance correlated with the decline in photosynthetic activity (Hawes et al., 2011b) and abundance of photosynthetic organisms in Legin Pond (Supplementary Figure S1) (Safi et al., 2012). It is feasible that these presumed chemoorganotrophs require particular dissolved organic carbon sources (DOC) for growth that are by-products from photosynthetic activity. These DOC sources would then be lost from the water column as photosynthesis ceased, resulting in a die-off due to lack of specific organic compounds. Further mass spectrometry, metatranscriptomics, and culture-based work would be needed to validate this hypothesis. On the other hand, the disappearance of *Actinomycetota* ASVs was correlated with the measured drop in water temperature over time; their disappearance is more likely to be related to the loss of sunlight than changes in temperature, assuming these species of *Aquiluna* are also photoheterotrophs.

It is unclear what is happening to these presumed aerobic heterotrophic bacterial groups over the winter, given that they are known to be highly abundant in the summer across annual sampling (Archer et al., 2014, 2016). We see three possibilities, as previously postulated (Priscu et al., 1998; Hawes et al., 2011b; Safi et al., 2012): they survive the winter at low abundances in some sort of inactive or dormant phase in the liquid phase; they become entrapped into the growing ice and melt out of the ice the following spring/summer; or they completely die out every winter and the ponds are re-seeded from elsewhere in the spring/summer. Further studies using culturing to determine whether these presumed heterotrophs are surviving in the liquid brine or ice are needed to differentiate between these hypotheses.

Also of note was the relatively large abundance of ASVs from *Thermoproteota* only in the second time point, especially the top-most sample (Figures 3D–F). These ASVs were all from the *Candidatus* Nitrosocosmicus genus, which are ammonia-oxidizing archaea (Lehtovirta-Morley et al., 2016), but ammonia concentrations were low in Legin Pond at this time point, peaking instead at lower depths in later time points (Safi et al., 2012). It could be that they are consuming the ammonia so rapidly that they draw it down to the low levels we detected (Martens-Habbena et al., 2009), but detailed metabolic and transcriptomic data would be needed to detect this type of activity.

We caution that many of the inferences we have made about potential functionality of ASVs in our data set are based on sequence similarity to previously cultured microorganisms. Similarity in 16S rRNA gene sequence does not necessarily imply that two species will share the same functional potential. This is especially true for enigmatic groups such as “*Nanoarchaeota*” where taxonomic classification below order was not possible for any ASVs in our data set. Moreover, the groups identified in our samples (*Ca.* Woesearchaeales and *Ca.* Pacearchaeales) remain uncultivated (Wurch et al., 2016; St. John et al., 2019), although other members of the broader “*Nanoarchaeota*” group have been cultivated (Heimerl et al., 2017; Hamm et al., 2019; Kato et al., 2022; Johnson et al., 2024). Thus, further characterization of the microbes in these communities using metagenomics, metatranscriptomics, and/or culturing is necessary to validate the findings presented here.

### Hypothesis 1.2: influence of depth on microbial community composition

Our hypothesis that depth would be an important factor correlated with community composition in this pond was supported. This was most apparent when examining depth combined with conductivity (chemocline vs. mixolimnion), since the volume of the pond changed over time and made depth a slippery variable. Previous results found that Legin Pond is highly chemically stratified by salinity, with lower microbial abundance in the mixolimnion and higher microbial abundance in the chemocline (Safi et al., 2012). Over time, this stratified structure tended to be compressed with the concentration of salts into the remaining liquid (Figure 2) (Hawes et al., 2011b; Webster-Brown et al., 2012). In the first few time points, we did observe some differences in microbial community composition between the chemocline and mixolimnion samples (Table 1, PERMANOVA results, Figures 3A,E,F). We observed different “*Nanoarchaeota*” ASVs in the upper samples and the most saline samples (Supplementary Figure S4). We also observed that *Pseudomonadota* ASVs, correlated with conductivity, were more prevalent in the lower samples (Figures 3E, 4, 5). Their presence in these higher conductivity samples may be a preference for more saline waters—as noted above, many of these genera have previously been isolated from marine or brackish lake samples. Or, as discussed above, it may be due to a preference for specific, photosynthetically-derived organic carbon compounds.

Past studies have consistently showed that conductivity is correlated with planktonic microbial diversity in non-maritime Antarctic meltwater ponds (Jungblut et al., 2005; Archer et al., 2014, 2015, 2016; Ramoneda et al., 2021; Kollár et al., 2023), which we also observed (Table 1 and Figure 4). However, we note that the strength of correlation between depth and microbial community, and conductivity and microbial community, was not strong in our data set (Mantel correlation coefficient = 0.2 and 0.32 for depth and conductivity, respectively), indicating that other environmental factors were also at work.

### Hypothesis 2: shift from autotrophy to heterotrophy

Previous studies reported that Legin Pond experienced a shift from photoautotrophy to heterotrophy with the onset of winter (Hawes et al., 2011b; Safi et al., 2012). Summer conditions in these ponds tend to favour the presence of photosynthetic organisms, as there is constant sunlight and the presence of liquid water, resulting in an explosion of photosynthetic organisms present, especially in the mats (Hawes et al., 1992, 2016). In the water column, photosynthetic organisms tend to be outcompeted throughout winter, autumn and spring (Bell and Laybourn-Parry, 1999) by organisms able to tolerate the harsh winter conditions. In support of our hypothesis, we did observe that all photosynthesis-related ASVs in our samples had almost completely disappeared by the final time point (Supplementary Figure S1). However, contrary to previous studies showing a broad diversity of filamentous *Cyanobacteriota* in the water column in Legin Pond (Safi et al., 2012), we only observed a single *Cyanobacteriota* ASV. One possible explanation to reconcile these results is primer bias: it may be that the primers used in this study are biased against these groups of *Cyanobacteriota*.

## Conclusion

There is a fascinating cascade of biological, physical, and chemical changes to the meltwater ponds of the McMurdo Ice shelf during the onset of winter. Our data provide insights into how the microbial communities in one of these ponds, Legin Pond, respond to these dramatic changes. We found that the communities present did have a strong response to the onset of winter, with a community primarily composed of enigmatic “*Nanoarchaeota*” becoming dominant at the end of the study. Depth of sample collection, especially when additionally considered with conductivity, also played an important role in this transition. Halo-sensitive and halo-tolerant microbes were separated at first by within-pond stratification, until the remaining liquid was freeze-concentrated all low-salinity adapted microbes were excluded. In the end, only non-phototrophic microbes adapted to higher conductivity conditions survived in the remaining liquid at the bottom of the pond before the ice completed its overwinter takeover.

## Data Availability

All R scripts used to analyze the data are available on Github at https://github.com/ThermophileResearchUnit/Extended-Season-manuscript. All sequence data is available in GenBank. 16S rRNA gene sequence data has been deposited as accessions PX370068–PX370363.

## Footnotes

1
https://www.inkscape.org/
