# Supplementary material for: Bacterioplankton dynamics during winter freezing in a meltwater pond near Bratina Island, Antarctica
Source: Front Microbiol. 2026 Jan 14;16:1707790. doi: 10.3389/fmicb.2025.1707790 (PMC12851595; doi:10.3389/fmicb.2025.1707790)
Supplement: Supplementary file 1 [file Data_Sheet_1.DOCX]

**Supplementary Material**

**Table S1** (found in a separate .csv file, Table S1.csv) Sample data for the data set with measured physicochemical parameters, dates of sampling, and corresponding ice thickness of Legin Pond at those time points.

**Table S2** (found in a separate .csv file, Table S2.csv) Tracking read counts through different stages of the DADA2 pipeline, including chimera removal.

**Table S3** (found in a separate .csv file, Table S3.csv) ASV data for the Legin Pond data set, including taxonomic information (NA = Unassigned), relative abundance in each sample (using rarefied data), and corresponding ASV DNA sequence. Low abundance (<10 reads across all samples) ASVs and Eukaryotes, mitochondria, and chloroplasts were removed prior to the creation of this table.

**Table S4** Correlations between environmental factors were measured using Spearmen’s correlation. Resulting *p*-values were adjusted for multiple tests using a Benjamini-Hochberg correction. DO: dissolved oxygen (mg/L). Dist.from.bottom: distance from pond sediment (cm).

| **Variable 1** | **Variable 2** | **Correlation Coefficient** | ***p*-value** | **Adjusted *p*-value** |
| --- | --- | --- | --- | --- |
| DO | Conductivity.mS | -0.0448 | 0.839 | 0.839 |
| DO | Dist.from.bottom | 0.1585 | 0.470 | 0.564 |
| DO | Temperature | 0.6770 | 3.88E-4 | 1.16E-3 |
| Conductivity.mS | Dist.from.bottom | -0.9225 | 3.85E-10 | 2.31E-09 |
| Conductivity.mS | Temperature | -0.5036 | 0.0143 | 0.0214 |
| Dist.from.bottom | Temperature | 0.5857 | 3.32E-3 | 6.64E-3 |


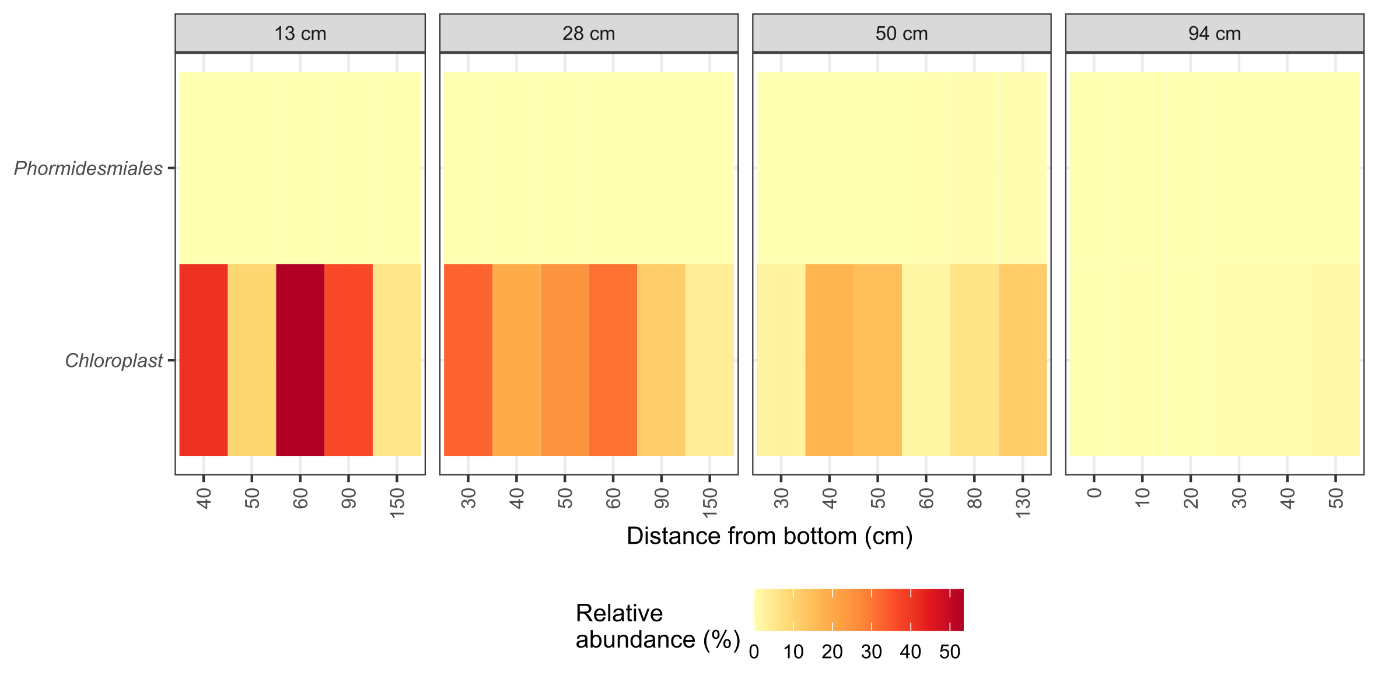


**Figure S1** Relative abundance of ASVs from photosynthetic groups, aggregated at the taxonomic level of order, separated by time point (top label denotes ice thickness at time of sampling) and depth (x-axis).

**
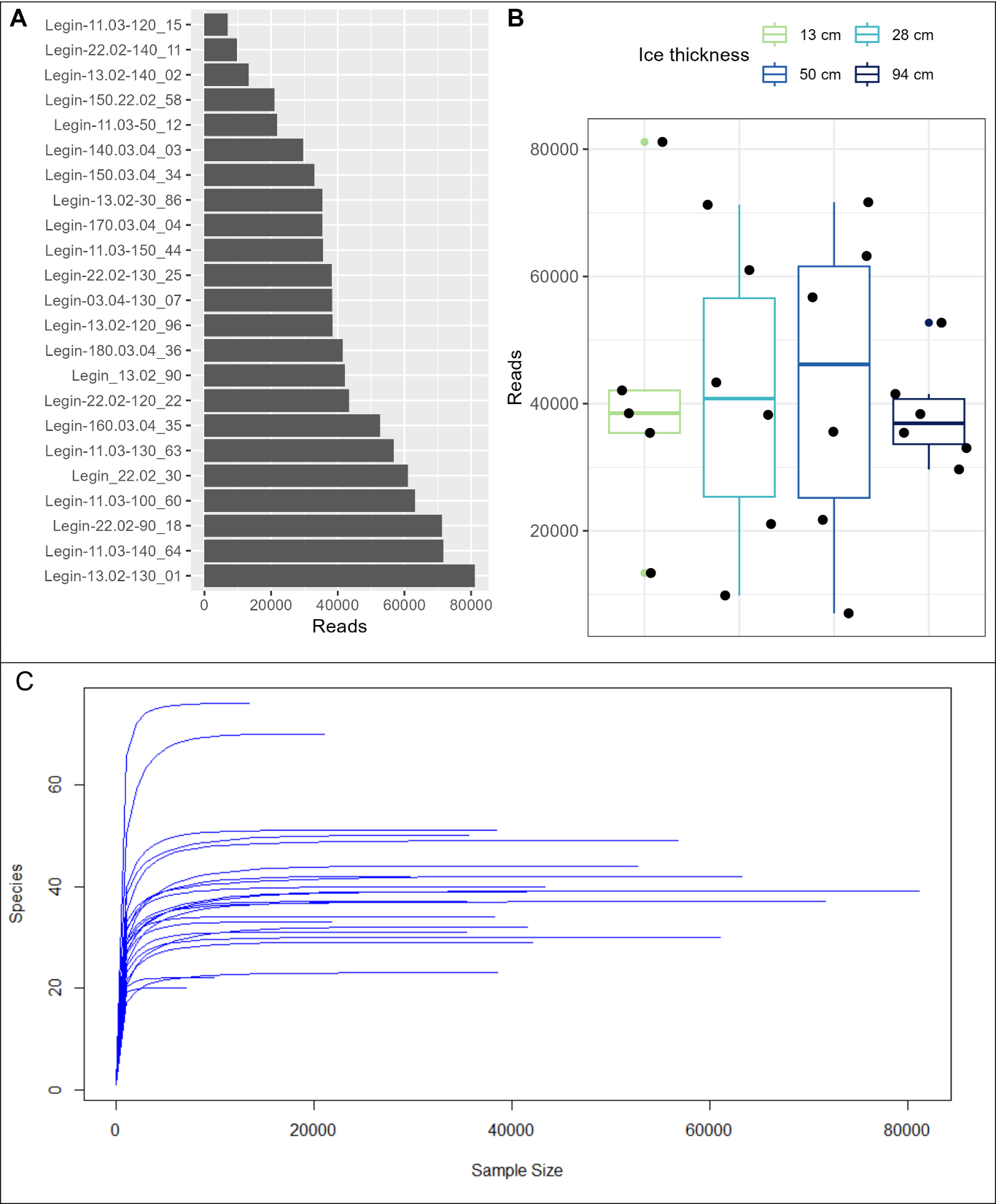
**

**Figure S2** Initial assessment of ASV data, after removing chimeras and eukaryote ASVs. (A) The number of sequencing reads varied substantially across different samples, but (B) the median read numbers were similar for all time points. (C) Rarefaction curves for all samples, with all samples reaching plateau.


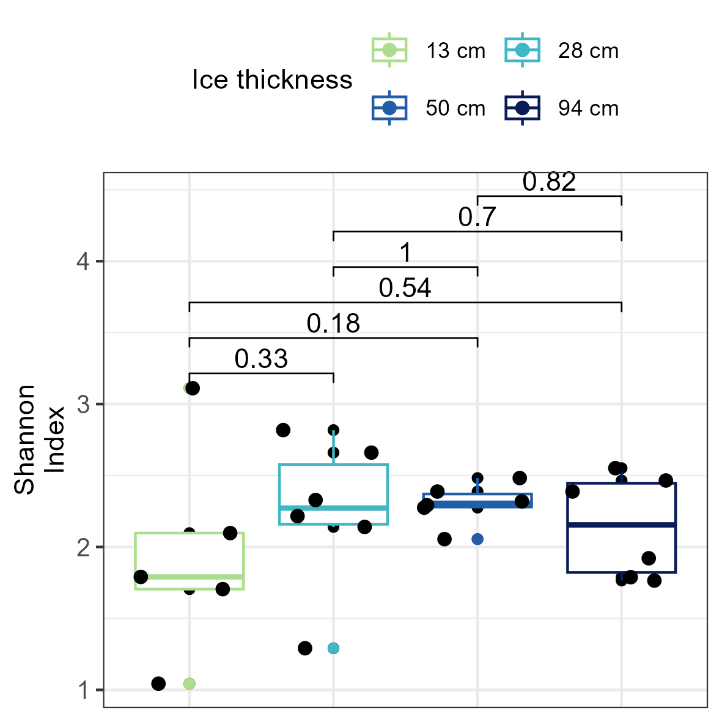


**Figure S3** Alpha diversity, measured using the Shannon index on rarefied ASV counts, comparisons between sampling time points. Given *p*-values are the result of pairwise Wilcox tests, adjusted for multiple testing via Benjamini-Hochberg adjustment.


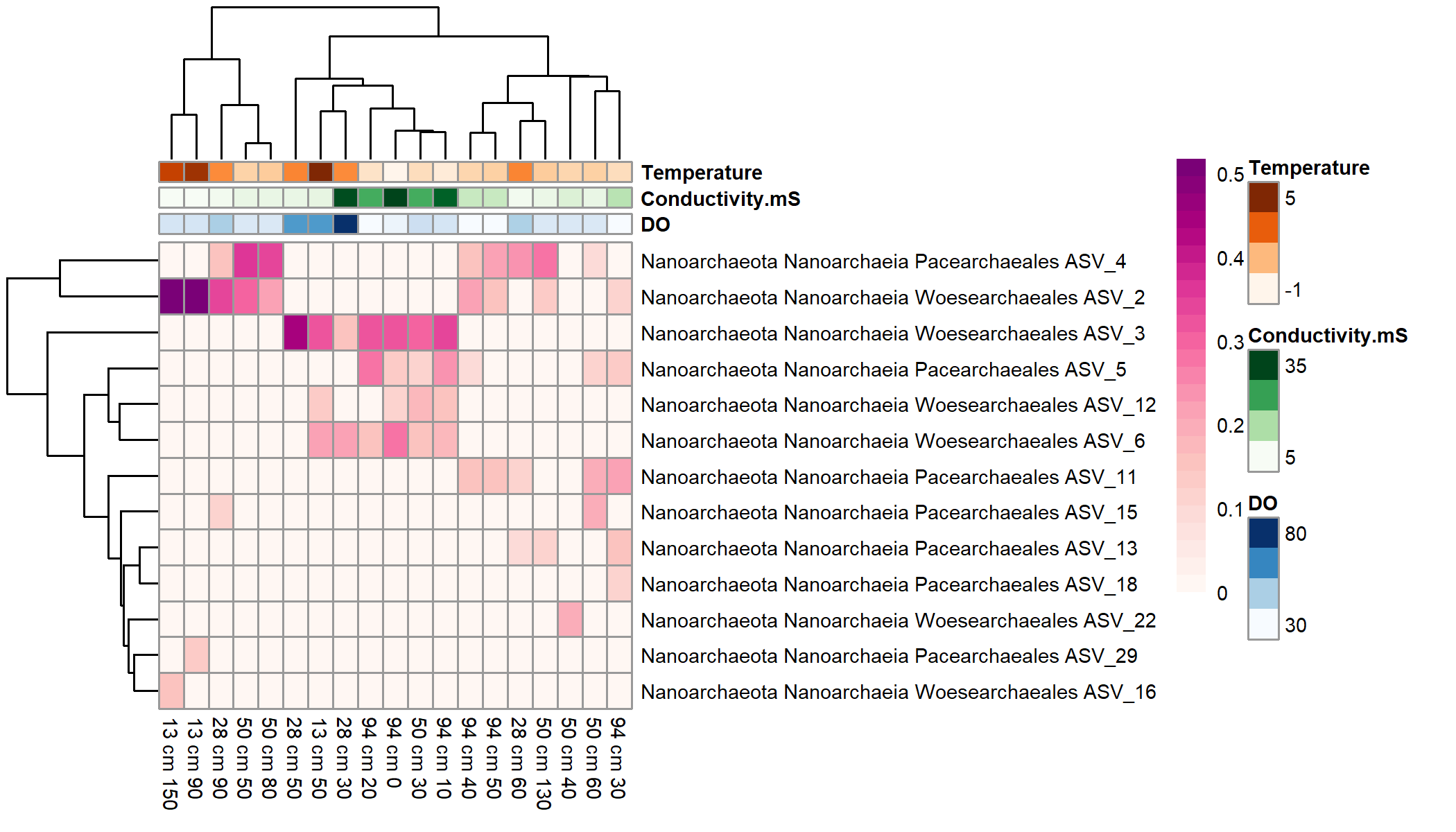


**Figure S4** Heatmap and dendrogram showing the relative abundance (%) of ‘*Nanoarchaeota’* ASVs with relative abundance >10%, clustered by similar abundance profiles. This resulted in four samples being dropped from this analysis due to a lack of ‘*Nanoarchaeota*’ ASVs that had relative abundance >10%. The dendrograms are indicative of similarity in abundance profiles and do not have any phylogenetic implications. Sample names given on the x-axis are in the format: “Ice thickness (cm)” “Distance from bottom (cm)”. DO: Dissolved oxygen (mg/L). Temperature is in °C. ASV names on the y-axis are in the format: Phylum Class Order ASV_number.
